# Effects of Toxic Organic Compounds on *Tenebrio molitor* and Its Parasite *Gregarina steini*

**DOI:** 10.3390/biology14050453

**Published:** 2025-04-23

**Authors:** Denis Rybalka, Viktor Brygadyrenko

**Affiliations:** 1 Department of Biodiversity and Ecology, Oles Honchar Dnipro National University, Nauky Av., 72, 49010 Dnipro, Ukraine; denisrybalka89@gmail.com; 2 Department of Parasitology and Veterinary and Sanitary Expertise, Dnipro State Agrarian and Economic University, Serhii Efremov St., 25, 49000 Dnipro, Ukraine

**Keywords:** *Tenebrio molitor*, *Gregarina steini*, parasitic systems, xylene, formaldehyde, aniline

## Abstract

Environmental pollution by toxic compounds affects various species of living organisms, their life cycles, and symbiotic relationships. This study investigated the host-parasite relationship between *Tenebrio molitor* Linnaeus, 1758 (Coleoptera, Tenebrionidae) and *Gregarina steini* Berthold, 1827 (Eugregarinorida, Gregarinida) under exposure to aniline, formaldehyde, and o-xylene at different concentrations. For laboratory studies, 480 larvae of *T. molitor* and five concentrations of the studied organic compounds were used. Groups of *T. molitor*, each consisting of ten individuals, were subjected to the tested compounds for 10 days following the initial weight measurement. We measured the body weight and survival rate of *T. molitor* and the number of *G. steini* in them to assess the impact of the tested organic compounds on the host-parasite relationship. For all the compounds studied, a decrease in body weight and high mortality were observed in *T. molitor* at high concentrations. The lethality of the studied organic compounds was concentration-dependent. o-Xylene showed low toxicity at lower concentrations (10.4 and 26.0 mg/kg of feed), with a survival rate of 93.3% and 86.7%, respectively. At a concentration of 10.4 mg/kg, no lethal cases in *T. molitor* were observed. At higher concentrations (78 and 104 mg/kg feed), *T. molitor* mortality significantly increased to 33.3% and 53.3%, respectively.

## Simple Summary

This study examined the effects of three common industrial chemicals—aniline, formaldehyde, and o-xylene—on mealworm larvae (*Tenebrio molitor*) and their internal parasites. Mealworms are used not only as a food source for humans and animals but also as model organisms to study the effects of pollution on small animals. In our experiment, we exposed larvae to different concentrations of these substances, assessing changes in body weight, survival rates, and parasite abundance. The results show that elevated concentrations of the studied compounds cause body weight loss and increased mortality among larvae, with aniline being the most harmful. At the same time, the number of parasites living in the larvae’s body remained virtually unchanged, even at high concentrations of pollutants. This indicates that although the larvae lost body weight and were more likely to die, their internal environment remained suitable for the parasites to survive. Our results highlight the threat that ubiquitous pollutants pose to even the smallest organisms. The findings highlight the need for further research, especially since *T. molitor* is considered a new and sustainable food source with a low carbon footprint and minimal space requirements for cultivation. Understanding how chemical pollution affects these insects is important for ensuring the safety and sustainability of future food systems, including the possibility of using mealworm larvae to sustain interplanetary space expeditions.

## 1. Introduction

Anthropogenic pollution is caused by chemical production, pharmaceuticals, construction, and the use of fossil fuels. Harmful compounds are mainly released through high-temperature combustion, explosions, and uncontrolled mining, contaminating water, soil, and air. The presence of persistent organic pollutants raises concern due to their ability to bioaccumulate in food chains, causing toxicity at higher trophic levels [1]. The long-term persistence of these pollutants in ecosystems intensifies their harmful effects, making efforts to remediate the situation complex and expensive [1].

The global production of aniline, xylene, and formaldehyde highlights their broad industrial significance. In 2022, manufacturers produced approximately 7 million tons of aniline, and forecasts expect it to grow by 5.3% annually until 2032. In 2019, experts assessed xylene production at 56 million tons. In 2022, the market valued xylene at USD 30.9 billion, and its compound annual growth rate (CAGR) is projected at 8.0% from 2023 to 2030. In 2022, manufacturers produced about 23 million tons of formaldehyde, with an anticipated annual growth rate of 3.4% through 2032 [2,3,4,5,6].

Xylene is an aromatic hydrocarbon that is typically found in petroleum and coal tar and is also formed during forest fires. Industries primarily use xylene as a solvent in printing, rubber, and leather production, as well as in the manufacture of paints and varnishes [7]. Xylene is a clear liquid with a sweet odor and a density of 0.86 g/cm^3^ at 20 °C [7]. The diversity of xylene isomers expands its applications across various sectors, including in the production of certain plastics [8]. There are two primary routes of xylene poisoning: inhalation and dermal contact. Absorption does not significantly differ between the three isomers. Once in the human body, approximately 95% of xylene is metabolized in the liver to methyl hippuric acid, which is then excreted in urine within 24 h, with only minor differences between isomers [9].

Contact with xylene induces oxidative stress in invertebrates, causing damage to DNA and cell membranes, inhibiting reproductive traits, and increasing mortality rates [10]. Studies on crustaceans show that xylene exposure disrupts molting processes and increases mortality [11]. Sublethal concentrations of xylene affect the feeding behavior of invertebrates, reducing their ability to acquire food [10]. Prolonged exposure to xylene leads to behavioral changes, including reduced mobility in invertebrates [11].

Aniline, an organic aromatic amine, contains a phenyl ring bonded to an amino group, which gives it unique properties and a wide range of applications. Aniline is a versatile organic compound with key characteristics that include a melting point of −6.3 °C and a boiling point of 184.1 °C under standard atmospheric pressure. It exhibits hydrophilic behavior, allowing it to mix with both water and organic solvents. These features determine its physical properties and its ability to form hydrogen bonds due to the presence of the amino group [12]. The industry widely uses aniline in the production of dyes, rubber, antioxidants, and pharmaceutical products, including the well-known analgesic paracetamol [13].

Aniline induces oxidative stress by generating free radicals, particularly in yeast, which leads to cytotoxic effects and genetic recombination. Brennan & Schiestl (1997) found that aniline and its metabolites, including 4-aminophenol and 2-aminophenol, are more toxic than aniline itself, causing oxidative DNA damage and cellular stress through the formation of reactive oxygen species. Additionally, aniline exposure is associated with increased toxicity in bacterial strains lacking antioxidant enzymes. Aniline’s cytotoxicity disrupts cellular defense systems, such as by reducing glutathione levels [14]. Its potential carcinogenicity is underscored by its ability to cause intrachromosomal recombination and chromosomal abnormalities in both microorganisms and mammalian cells [15,16].

Studies on the toxic effects of aniline have shown a significant impact on microbial communities and soil health. Abe (2001) found that aniline derivatives severely disrupted embryonic development and caused other abnormalities in *Daphnia magna* Straus, 1820, demonstrating acute toxicity even at relatively low concentrations. The delay in embryonic development observed at low concentrations of aniline suggests potential long-term consequences for population dynamics [17]. Franco (1984) reported dose-dependent toxicity of aniline for chironomid larvae: LC_50_ = 287 mg/L for *Tanypus punctipennis* Meigen, 1818 and LC_50_ = 442 mg/L for *Einfeldia natchitocheae* (Sublette, 1964). Aniline exposure caused behavioral abnormalities, including reduced mobility and increased mortality [18].

Formaldehyde is a substance widely used across many industrial sectors, including the production of resins, adhesives, textiles, and paper. Due to its antimicrobial properties, it also serves as a laboratory preservative and sterilizing agent. The internal presence of formaldehyde plays a vital role in cellular metabolism by supporting the synthesis of essential biomolecules, including purines and specific amino acids [19]. However, due to its water solubility and chemical reactivity, formaldehyde can rapidly spread through various biological tissues after absorption. The body primarily metabolizes it through reactions with glutathione, resulting in intermediate compounds such as hydroxymethylglutathione, which are further processed and excreted as formic acid [19].

Formaldehyde exhibits a range of toxic effects due to its high reactivity with nucleophilic groups in proteins, DNA, and RNA, leading to genotoxic and carcinogenic outcomes [19]. The International Agency for Research on Cancer (IARC) classifies formaldehyde as a human carcinogen, with prolonged exposure linked to cancers of the upper respiratory tract. Bernardini (2020), through in vitro studies on aquatic invertebrates, demonstrated that formaldehyde exposure induces oxidative stress, mitochondrial dysfunction, and apoptosis in sensitive cells. Formaldehyde exposure is also associated with DNA-protein cross-linking and an increased frequency of micronuclei [20].

Chénier (2003) reports a high sensitivity of freshwater invertebrates to formaldehyde: ostracods of the genus *Cypridopsis* showed an EC_50_ (immobilization) value of 0.36 mg/L after 96 h of exposure. For terrestrial invertebrates, nematodes in peat were eradicated by treatment with a formaldehyde solution at a concentration of 370 g/L and a dose of 179 mL/m^3^ [21].

*Tenebrio molitor* Linnaeus, 1758 is known as one of the most important insects in the world in terms of breeding and trade. *Tenebrio molitor* is often used as an environmental bioindicator and is commonly used in laboratory experiments [22]. By consuming food products, *T. molitor* reduces its mass and lowers its nutritional value [23]. In addition to overall weight loss, stored products become contaminated with dead insects and their excrement.

*Tenebrio molitor* larvae are gaining popularity as a novel food product, as they are recognized as a sustainable source of protein, amino acids, vitamins, and minerals for both humans and animals. This aligns with global trends in the search for new and sustainable food sources, as farmed insects typically require less land and fewer resources compared to conventional crop or livestock farming [24].

Plastic materials are generally resistant to degradation and long-lasting. However, there are reports that *T. molitor* and other storage pests consume products packaged in plastic. They chew through hard plastics using their mandibles. *Tenebrio molitor* and other insects can live on a plastic-polymer diet without population decline [25]. There is a correlation between plastic consumption behavior and the digestive activity of gut microorganisms, which facilitate the digestion process [25].

Unicellular gregarines most often choose arthropods as hosts; in the case of *T. molitor*, this may lead to an unpredictable reduction in the beetle’s lifespan. Invertebrate pathogens are more frequently observed in mass-rearing systems, often due to overcrowding and stress—for example, microsporidian infections in mites bred commercially for pest control [26].

In this regard, the aim of our study is to assess and compare the effects of aniline, formaldehyde, and o-xylene on the body mass and mortality of *Tenebrio molitor* larvae, as well as the parasitic load of *Gregarina steini*.

## 2. Materials and Methods

*Tenebrio molitor* used for the experiments were reared in the laboratory. Larvae of this species were kept in transparent plastic containers (40 × 30 × 20 cm) at a temperature of 21–23 °C, relative humidity of 40–50%, and indirect sunlight. All insects received the same feed: pressed oat flakes from a single batch (produced in 2024). The manufacturer (LLC “Skvyra Bakery Plant”, Skvyra, Ukraine) stated that no pesticides were used during oat cultivation. Crushed oat grains were evenly distributed in the container (occupying ⅔ of the container’s volume) [23,27,28]. We used cabbage leaves as a water source for *T. molitor*, placing them on the surface of the feed substrate after cutting them into 1 × 1 cm squares. We allocated 0.3 g of cabbage leaf per larva in each container. Cabbage leaves were replaced every 5 days to prevent mold. Humidity, temperature, and light were the same for all *T. molitor*. They had equal access to food and equal chances of being infected by parasites.

We selected 480 larvae for the experiment. The larvae were placed in groups of 10 individuals in separate transparent plastic containers with a volume of 500 mL, each containing 100 g of crushed oat grains. All chemicals were purchased from Sigma Aldrich unless otherwise stated (Sigma-Aldrich Chemie GmbH, 82024 Taufkirchen, Germany). To evaluate the effects of aniline, formaldehyde, and o-xylene, on *T. molitor* larvae and their parasites. The concentration ranges used in this study—10.4, 26, 52, 78, and 104 mg/kg of feed substrate—were selected to model both environmentally relevant exposure levels and worst-case contamination scenarios. For o-xylene, the Canadian Soil Quality Guidelines [29] establish protective thresholds of 2.4–11 mg/kg, with adverse effects on soil invertebrates such as *Eisenia foetida* observed at concentrations as low as 8 mg/kg, and significant mortality occurring at 78 mg/kg, which supports our use of higher levels for ecotoxicological testing. Although formaldehyde is highly volatile and not expected to persist in soil, its acute release in industrial or military contexts justifies its inclusion at elevated concentrations to simulate short-term exposures, as noted in the U.S. EPA’s [30] environmental exposure assessment. Regarding aniline and its chlorinated derivatives, environmental concentrations in water bodies range from <0.00006 to 67 µg/L, but toxicity in aquatic organisms has been reported at thresholds between 100–500 µg/L and higher. Rebelo et al. (2023) emphasized the persistence and bioaccumulation potential of compounds like 4-chloroaniline and 3,4-dichloroaniline, as well as their disruptive effects on reproduction, oxidative stress responses, and organism behavior at sublethal concentrations [31]. Although our concentrations exceed natural background levels, they fall within the range used in ecotoxicology to simulate contaminated substrates and assess sub-individual and population-level effects. For each pollutant concentration, we used three groups of *T. molitor*. The substances were evenly introduced into the feed substrate using a pipette at a uniform depth of ~50 mm from the surface. The substrate was then thoroughly mixed, and the larvae were placed into it. Each larva was marked with a number using a marker.

The larvae were exposed to the chemicals for 10 days. Control groups were kept under the same conditions but without exposure to additional substances. We placed a cotton pad at the bottom of each plastic container to absorb excess moisture. On top of the feed substrate, we placed 3 g of cabbage leaves, pre-cut into 1 × 1 cm squares. The experiment was conducted under uniform conditions, including sunlight, relative humidity of 40–50%, and temperature of 21–23 °C. After five days of exposure to the test substances, we weighed each *T. molitor* specimen individually to assess changes in body mass.

**Figure 1:**
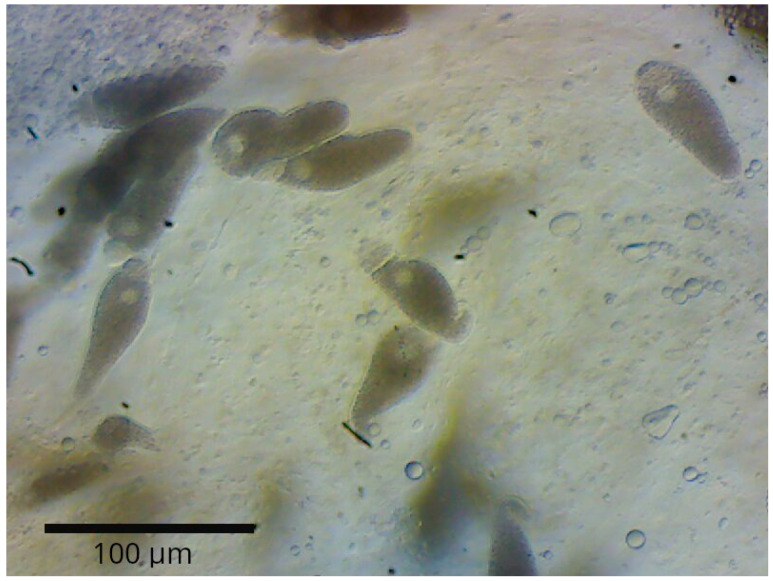
*Gregarina steini* in the gut of *T. molitor* larvae: bar—100 µm.

After completing the experiment, we removed the intestines of *T. molitor*. The midgut (from the stomach to the Malpighian tubules) was placed on a clean microscope slide in a drop of physiological saline (0.9% NaCl solution) to prevent drying and to facilitate the observation of gregarines. Using a scalpel, we opened the intestine by making 12 transverse incisions. Within three minutes of dissection, we analyzed the larval midgut by systematically scanning the slide using stage movement under a microscope [32,33]. We excluded immature gregarines (gamonts attached to the gut wall via the epimerite) from the count. According to established classifications, once the epimerite is reduced, the gregarine transitions into a freely motile trophozoite stage within the host’s gut [34]. Only trophozoites released from the midgut during standardized compression with a coverslip were counted. We acknowledge that a small proportion of trophozoites may remain inside the gut; however, we ensured consistency across all samples by applying the same dissection protocol, including standardized incisions, equal volumes of 0.9% NaCl solution, and uniform pressure applied via coverslip. This allowed us to maintain comparability across experimental treatments. We found and identified *Gregarina steini* (Figure 1) based on its morphological characteristics [34]. We refined the identification of *G. steini* using the publications of Clopton & Janovy [32,33], and Desportes & Schrével [35].

We conducted all statistical analyses using R version 4.4.2 (R Core Team, 2024) in RStudio version 2024.12.0+467. We used the following R packages: dplyr (version 1.1.4), ggplot2 (version 3.5.1), and multcompView (version 0.1-10) for post hoc statistical analysis. We performed a one-way analysis of variance (ANOVA) and Tukey’s HSD test to identify pairwise differences between pollutant concentrations, *G. steini* abundance, and changes in *T. molitor* body mass. We considered differences statistically significant at *p* < 0.05.

## 3. Results

Exposure to aniline, formaldehyde, and o-xylene, increased mortality in *T. molitor* larvae. At lower concentrations (10.4 and 26 mg/kg), the larvae showed relatively high survival rates (93.3% for o-xylene and formaldehyde at 10.4 mg/kg, 86.7% at 26 mg/kg). Increasing the concentration significantly reduced survival: o-xylene caused 53.3% mortality at 104 mg/kg, formaldehyde caused 60.0% mortality at the same concentration, and aniline showed the highest toxicity—66.7% mortality at 104 mg/kg.

Larval mortality in *T. molitor* significantly increased with higher concentrations of organic substances (Table 1). At 52 mg/kg, over 20% of the beetle larvae died. Mortality exceeded 50% at 104 mg/kg of aniline, formaldehyde, or o-xylene.

In the control group, all larvae survived, resulting in an imago image transformation rate of 16.7%. The absence of mortality in the control confirmed that the experimental conditions did not influence the results. These findings indicate that aniline is the most toxic compound, followed by formaldehyde and then o-xylene (Table 1).

**Figure 2:**
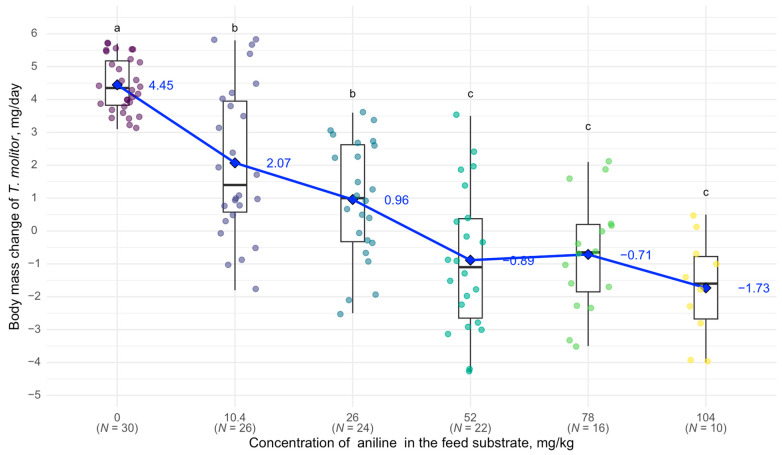
Correlation between *T. molitor* body weight and aniline concentration: black horizontal line—median, blue diamond—mean, lower and upper boundaries of the rectangle—I and III quartiles, the black vertical line above the box represents the range from the third quartile to the maximum value, and the black vertical line below the box represents the range from the first quartile to the minimum value, the dots represent individual *T. molitor* specimens, different letters above the boxes indicate statistically significant differences (*p* < 0.05) between populations according to Tukey’s HSD test, the numbers next to the blue line indicate the mean value.

At a concentration of 10.4 mg/kg of aniline in the feed substrate, body mass changes began to differ significantly from the control group: in the control, *T. molitor* larvae gained an average of 4.45 mg per day, while at 10.4 mg/kg, they gained only 2.07 mg per day. At 52 mg/kg, *T. molitor* larvae began to lose body mass during the experiment—on average, −0.89 mg per day. At 104 mg/kg, the average body mass loss was 1.73 mg per day, with 66.7% mortality (Figure 2).

**Figure 3:**
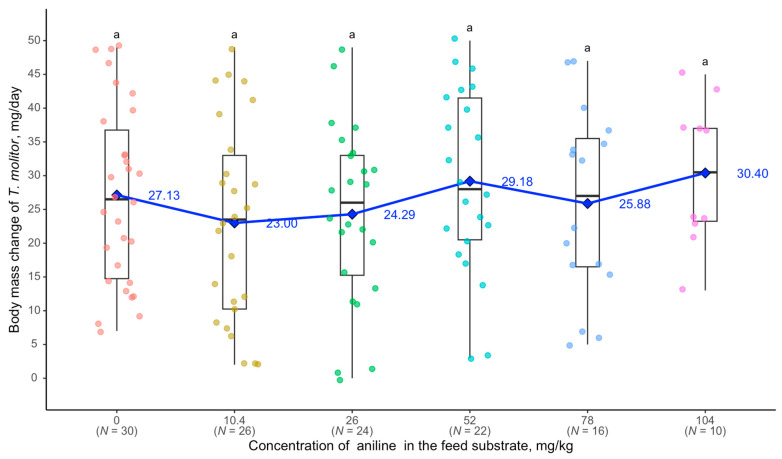
Relationship between *G. steini* abundance in *T. molitor* and aniline concentration: see Figure 2.

Differences between various concentrations of aniline, including the control group, were statistically insignificant. The regression equation y = 0.0420x + 24.6001 (R^2^ = 0.010) indicates a very weak relationship between the concentration of aniline in the feed substrate and the number of *G. steini* in *T. molitor* larvae (Figure 3).

**Figure 4:**
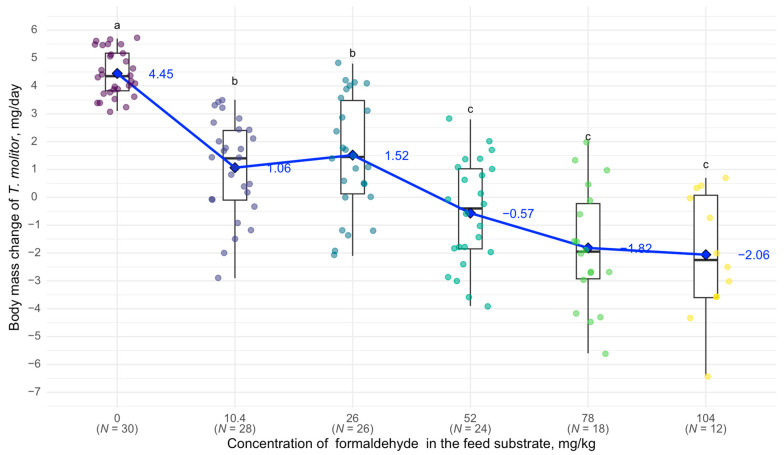
Relationship between body weight of *T. molitor* and formaldehyde concentration: see Figure 2.

At a concentration of 10.4 mg/kg of formaldehyde in the feed substrate, body mass changes began to differ significantly from the control group: in the control, *T. molitor* larvae gained an average of 4.45 mg per day, whereas, at 10.4 mg/kg, they gained only 1.06 mg per day. At 52 mg/kg, the larvae began to lose body mass during the experiment (an average of −0.57 mg per day). At 104 mg/kg, *T. molitor* showed an average body mass loss of 2.06 mg per day and 60% mortality (Figure 4).

**Figure 5:**
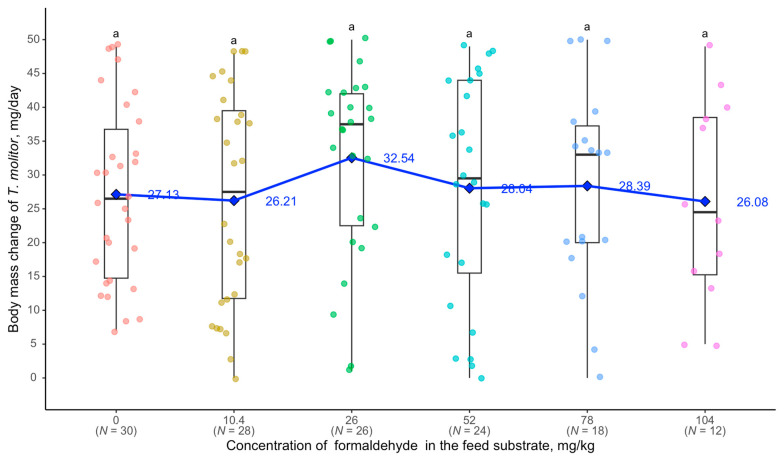
Relationship between *G. steini* abundance in *T. molitor* larvae and formaldehyde concentration: see Figure 2.

The relationship between *G. steini* abundance in *T. molitor* and formaldehyde concentration did not show a clear trend. There were fluctuations in parasite counts without statistically significant differences between concentrations. The regression equation confirms this: y = −0.0013x + 28.2410 (R^2^ = 8.0 × 10^−6^, Figure 5).

**Figure 6:**
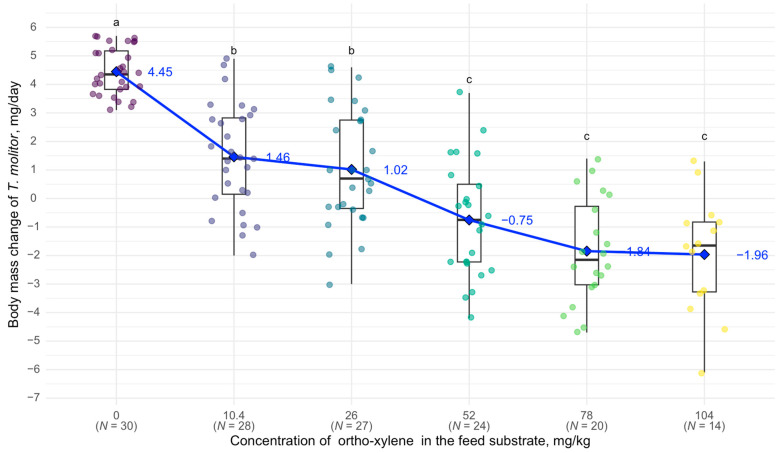
Relationship between body weight of *T. molitor* and o-xylene concentration: see Figure 2.

At a concentration of 10.4 mg/kg of o-xylene in the feed substrate, body mass changes began to differ significantly from the control group: in the control, *T. molitor* larvae gained 4.45 mg per day, whereas, at 10.4 mg/kg, they gained only 1.46 mg per day. At 52 mg/kg, the larvae began to lose body mass during the experiment (an average of −0.75 mg per day). At 104 mg/kg, *T. molitor* showed an average body mass loss of 1.96 mg per day and 53.5% mortality (Figure 6).

**Figure 7:**
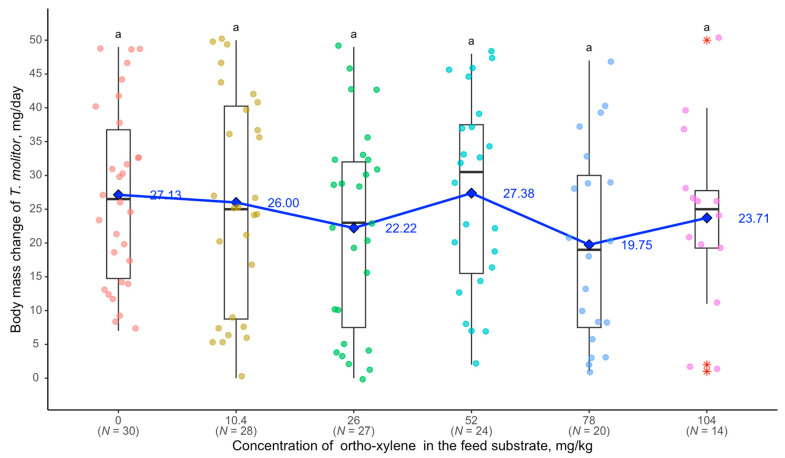
Relationship between *G. steini* abundance in *T. molitor* larvae and o-xylene concentration: see Figure 2. Red asterisks representing outliers, values that deviate markedly from the interquartile range (1.5 * IQR).

The absence of statistically significant differences suggests that the observed changes were due to variability rather than a strong effect of o-xylene. The regression equation supports this: y = −0.0419x + 26.1980 (R^2^ = 0.0096, Figure 7).

## 4. Discussion

Our study showed that exposure to organic pollutants—specifically aniline, formaldehyde, and o-xylene—alters the body mass of *T. molitor* larvae. We found a clear relationship between pollutant concentration and body mass: as the concentration increased, larval mass decreased. However, the abundance of *G. steini* did not respond consistently to increasing pollutant concentrations, as indicated by low correlation coefficients (R values) indicating the absence of a statistically meaningful relationship between pollutant concentration and parasitic load. Although no data were found regarding environmental effects on *Gregarina steini*, studies on the related species *G. polymorpha* have shown that dietary inclusion of various organic xenobiotics did not significantly alter parasitic load in *T. molitor* larvae [36]. Recent studies have demonstrated that *T. molitor* is a promising model for evaluating the acute toxicity and metabolic processing of xenobiotics [37]. These findings align with our observations of dose-dependent changes in body mass and mortality, further validating *T. molitor* as a sensitive indicator organism for pollutant-induced stress.

Reinhard (2010) noted that invertebrates lack a single organ responsible for taste (like the human tongue), but their mouthparts contain gustatory sensilla that fulfill this function. In many beetles, these are located on the maxillary and labial palps. Additionally, insects have taste receptors inside the oral cavity and along the esophagus. We can assume that the feed substrate did not lose its attractive taste for *T. molitor* and continued to be ingested. Even at high concentrations, the number of *G. steini* in *T. molitor* larvae did not differ from the control group, but the toxic effects on the host’s physiology were evident, which in turn led to decreased larval body mass [38].

Aniline demonstrated the highest toxicity among the three compounds, causing greater mortality even at low concentrations. This corresponds with previous studies [14,39], which show that aniline induces oxidative stress and disrupts cellular integrity. O-xylene and formaldehyde also exhibit toxic effects, but their impact on larvae was weaker at lower concentrations [10,40]. Nevertheless, in groups exposed to low pollutant concentrations, we observed increases in body mass, which may indicate an adaptive response of larvae to pollutant exposure. These findings are useful for future studies, especially in the context of *T. molitor* population control, as the species is considered a pest in agroecosystems.

The number of *G. steini* in larval intestines varied depending on pollutant concentration; however, we did not establish a consistent relationship between infection levels and concentration. At lower concentrations, we observed more gregarines, whereas at higher concentrations their numbers decreased. This may be explained by the fact that pollutants alter the host’s internal environment, making it less suitable for parasite survival [36,41,42].

Larvae with greater body mass tended to have higher numbers of gregarines. This supports the assumption that gregarine infection is associated with active food intake, where the food contains parasite oocysts. However, even with high infection intensity, we did not observe a significant negative effect of gregarines on body mass accumulation or larval viability, which aligns with studies on the symbiosis between *G. steini* and *T. molitor* [43].

The study results confirmed that larval mortality depends on pollutant concentration. In the control group, no cases of *T. molitor* death were recorded. Meanwhile, at 102 mg/kg, mortality reached 53.3% for o-xylene, 60.0% for formaldehyde, and 66.7% for aniline. These mortality rates confirm the principle of dose-dependent toxicity of pollutants [44,45].

It should be noted that all experiments were conducted at a temperature of 21–23 °C. Temperature conditions can significantly influence the concentration of organic compounds and, accordingly, their toxic effects. Future research should include testing the impact of pollutants under different temperature conditions and assessing the combined effects of two or more pollutants. This will help better understand the influence of toxic substances on *T. molitor* and its symbiosis with gregarines.

*Tenebrio molitor* is rich in protein, essential fatty acids, and trace elements, which makes it an efficient and sustainable nutritional source for long-term space missions. Kok & van Huis (2021) proposed using *T. molitor* as a regular food source on spacecraft, enabling daily harvesting to support the crew for extended periods. *Tenebrio molitor* can be reared on organic waste, converting it into edible protein, which minimizes resource consumption [46]. This makes them a valuable component in closed ecosystems designed for space settlements. Considering this, further research on laboratory cultures of this insect species is necessary.

## 5. Conclusions

The results of our study highlight the significant ecological threats posed by the introduction of the tested pollutants into the environment of *T. molitor* larvae. We observed increased larval mortality at high concentrations of the pollutants used in the study. O-xylene and formaldehyde demonstrated moderate toxicity to *T. molitor* at lower concentrations, while aniline proved to be the most toxic compound, causing the highest mortality even at minimal concentrations.

At concentrations of 52 mg/kg and higher, the majority of *T. molitor* larvae began to lose body mass during the experiment for all tested compounds. The greatest body mass changes were recorded at the highest concentrations: on average 1.63, 2.20, and 1.58 mg of body mass loss per day for aniline, formaldehyde, and o-xylene, respectively. We conclude that T. molitor lost on average more body mass under the influence of formaldehyde (2.20 mg/day) compared to aniline (1.63 mg/day) and o-xylene (1.58 mg/day) at a concentration of 104 mg/kg of these toxicants in the feed substrate. However, larval mortality was not the highest: 60.0% with formaldehyde, 66.7% with aniline, and 53.3% with o-xylene. We found no statistically significant differences in the number of *G. steini* depending on the concentration of aniline, formaldehyde, or o-xylene in the *T. molitor* feed substrate.

## Figures and Tables

**Table 1:** Mortality of *T. molitor* larvae in a 10-day laboratory experiment after exposure to different concentrations of organic pollutants.

Substance	Concentration of the Studied Compounds, mg/kg						*χ* ^2^	*p*
	0	10.4	26	52	78	104		
Aniline	0	13.0	20.0	26.7	46.7	66.7	172.89	<0.001
Formaldehyde	0	6.7	13.3	20.0	40.0	60.0	104.52	<0.001
O-xylene	0	6.7	13.3	20.0	33.3	53.3	75.43	<0.001

## Data Availability

Data supporting this study are openly available at https://github.com/RybalkaDenis/biology-14-00453 (accessed on 20 March 2025).
